# The pleasure of multiple images

**DOI:** 10.3758/s13414-020-02175-z

**Published:** 2020-11-17

**Authors:** Aenne A. Brielmann, Denis G. Pelli

**Affiliations:** 1 Psychology Department, New York University, New York, NY USA; 2 Max Planck Institute for Biological Cybernetics, Tübingen, Germany; 3 Center for Neural Science, New York University, New York, NY USA

**Keywords:** Precuing, Scene perception, Object recognition

## Abstract

How many pleasures can you track? In a previous study, we showed that people can simultaneously track the pleasure they experience from two images. Here, we push further, probing the individual and combined pleasures felt from seeing four images in one glimpse. Participants (*N* = 25) viewed 36 images spanning the entire range of pleasure. Each trial presented an array of four images, one in each quadrant of the screen, for 200 ms. On 80% of the trials, a central line cue pointed, randomly, at some screen corner either before (precue) or after (postcue) the images were shown. The cue indicated which image (the target) to rate while ignoring the others (distractors). On the other 20% of trials, an X cue requested a rating of the combined pleasure of all four images. Later, for baseline reference, we obtained a single-pleasure rating for each image shown alone. When precued, participants faithfully reported the pleasure of the target. When postcued, however, the mean ratings of images that are intensely pleasurable when seen alone (pleasure >4.5 on a 1–9 scale) dropped below baseline. Regardless of cue timing, the rating of the combined pleasure of four images was a linear transform of the average baseline pleasures of all four images. Thus, while people can faithfully track two pleasures, they cannot track four. Instead, the pleasure of otherwise above-medium-pleasure images is diminished, mimicking the effect of a distracting task.

**Supplementary Information:**

The online version of this article (10.3758/s13414-020-02175-z) contains supplementary material, which is available to authorized users.

In everyday life, we often evaluate how much pleasure we feel from one object among many or the combined pleasure of several objects. Take, for instance, going out to a movie. Most of us feel confident that we can rate how much we enjoyed the movie independent of the comfort of the seats and the taste of the popcorn. At the same time, we also feel able to rate the movie night experience as a whole, taking all its different aspects into account. However, from a scientific standpoint, we do not know whether people can indeed keep track of more than two pleasures independently. And we do not know how they combine the pleasure of more than two stimuli into an overall pleasure rating.

## A bias toward the average when reporting one item in an array

When several stimuli are shown at once, people’s ratings of one item’s properties, like size or color, are biased toward the average of the set (e.g., Brady & Alvarez, 2011; Haberman & Whitney, 2009; Maule, Witzel, & Franklin, 2014). Such a bias has been shown for simple features like the size of a dot (Brady & Alvarez, 2011; Corbett, 2017), or the color of a patch (Maule et al., 2014), and also for more complex judgments like facial emotions (Haberman & Whitney, 2009). For tight arrays in peripheral vision, this kind of averaging is not merely possible, but compulsory (Parkes, Lund, Angelucci, Solomon, & Morgan, 2001).

In contrast, we found that people give unbiased ratings of the pleasure they feel from one out of two images (Brielmann & Pelli, 2020). Studies of the reporting of a feature of one item among others typically use an array of more than two items (but see Maule et al., 2014). One explanation for a bias toward the mean is that it is a strategy for coping with the limited capacity of working memory: People can only store representations of a few items, and if they are cued to recall the feature value of a nonstored item, they report the average across stored items (Zhang & Luck, 2008). A set size of two seems unlikely to exhaust working memory capacity, so a bias toward the mean might appear only with more items.

One recent study found that people’s pleasure rating of one scene among three others is biased toward the mean pleasure of the presented scenes (Alwis & Haberman, 2020). That study differs from ours in using a long image presentation (1.5 s), time enough for more than three glimpses, whereas we allowed time (0.2 s) for only a single glimpse. Their observers might have serially sampled the three scene pleasures, one by one; our observers had to sample all four images in one glimpse. Thus, it is yet unclear how much pleasure observers get from one of several images without serial attention to each. The current study aims to answer this question.

## People can report the average of an array of items

When confronted with several objects simultaneously, we often want to assess not just how much we enjoy one of them but also their combined effect. This combined evaluation represents a summary statistic of the entire array of objects. When it comes to judgment of object properties, the formation of such a summary statistic is known as *ensemble perception*. Most research on ensemble perception has focused on reporting averages.

People make unbiased reports of the average feature value for a set of items for many kinds of feature, like orientation (e.g., Parkes et al., 2001), size (de Fockert & Marchant, 2008), position (Alvarez & Oliva, 2008), motion (e.g., Watamaniuk, 1993), and number (e.g., Burr & Ross, 2008), as well as facial identity (e.g., Neumann, Schweinberger, & Burton, 2013) and emotion (Fischer & Whitney, 2011; Haberman & Whitney, 2007). When only two images are presented, participants asked about the combined pleasure reported the average across the two images (Brielmann & Pelli, 2020). Whether or not people truly perceive the average of an object property (like size) or base the reported average on a selective sample of the entire array is controversial (e.g., Myczek & Simons, 2008).

Another phenomenon in ensemble perception worth mentioning is that the report variance is conserved across set size (e.g., Ariely, 2001; Chong & Treisman 2005) all the way down to one item (Allik, Toom, Raidvee, Averin, & Kreegipuu, 2013; Brielmann & Pelli, 2020). The finding that increased set size fails to decrease the variability of average reports suggests that the variance of reporting is limited by a late noise that arises after combining the members of the ensemble.

The average is often a useful summary statistic—for instance, when judging the emotion of a crowd. However, it is not clear whether the average is also the summary statistic used to judge combined pleasure—for instance, when we judge how pleasantly a room is decorated with various pieces of furniture. It might be that combined pleasure ratings are not an average, but mostly a reflection of the most or least pleasant object in view. When presented with two images, people do report their combined pleasure as the average of the two images’ individual pleasures (Brielmann & Pelli, 2020). However, performance with only two images cannot distinguish all the competing accounts. Here, we increase the number of images to four, which allows us to test several alternative models of pleasure combination across multiple items. The use of a larger array also provides comparability to previous studies of object property ratings.

## Current study

Here, we test whether the reported pleasure from an array of four images behaves like reports of objective object properties. We test whether the reporting of subjectively felt pleasure from one image among four is biased toward the mean, like the reporting of objective object properties. We also test whether people report the *combined* felt pleasure of four images as the mean across felt pleasures for the displayed images. While our previous study (Brielmann & Pelli, 2019) laid the groundwork for the current one, the use of only two images at a time did not allow us to distinguish mean-bias effects from direct contrast or assimilation effects. Here, we use the same procedure, to ease comparison, but now we can explicitly test whether subjective pleasure reporting is biased toward the mean, like the reporting of objective object properties. Thus, we probe the limits of how many pleasures people can track, and we work to incorporate subjective pleasure into ensemble perception.

## METHODS

### Participants

The observers in this study were 25 undergraduate students at NYU. We did not record the age or gender of our participants because we had no hypotheses relating to this information, and collected only necessary personal information in line with the ethics board guidelines. All participants were 18 years or older. All participants gave written informed consent according to the declaration of Helsinki. Approval was obtained from the NYU University Committee on Activities Involving Human Subjects (UCAIHS; IRB-FY2016-404). Each participant received course credit as compensation.

### Stimuli

We used the same 36 images from the OASIS database as in a previous study investigating whether people can track the pleasure of two images (Brielmann & Pelli, 2020). The selection criteria and rationale are discussed in depth in that report. The 36 images uniformly span the complete range of the 1–7 beauty scale, based on average ratings in an independent study (Brielmann & Pelli, 2019). Four images with average beauty ratings from the middle of the distribution were presented on training trials. The four training images were not part of the 36 images used in the main experiment.

## Procedure

Apart from the number of images and combined-pleasure trials, all procedures were identical to our previous study (Brielmann & Pelli, 2020).

Participants viewed the images on a 27-in. iMac Retina display (58.2 cm × 36.4 cm, set to 1,600 px × 900 px) from a distance of approximately 1 m (so visual angles specified below are approximate). When white, the screen is 500 cd/m^2^. The room was normally lit.

**Fig. 1:**
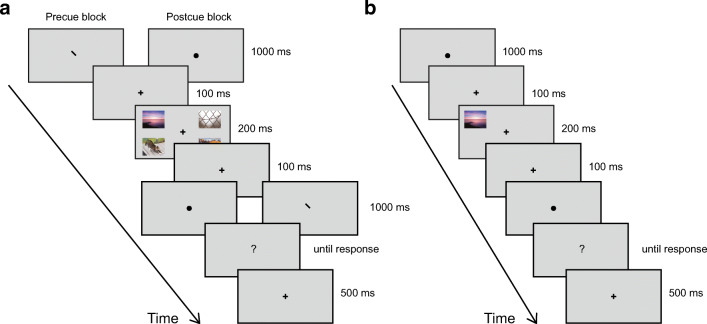
Timeline for a typical trial of the main experiment (**a**) and baseline rating (**b**). **a** During the main experiment, participants were either cued by a line to rate the image in the screen corner pointed to by the line, or cued by an X to rate the combined pleasure of all four images. Each line cue lies along the path from fixation to a screen corner. The X (not shown) is the combination of all four possible line cues

Trial timelines for the different trial types are illustrated in Fig. 1. A fixation cross of 20 px (about 0.4 deg) width and height was always present in the center of the screen, except while it was replaced by a cue or question mark. Participants were instructed to maintain central fixation whenever looking at the screen. This is a standard instruction in a variety of vision research paradigms, and it is commonly accepted that fixation of a central fixation cross on a blank field is easy, and that observers do this faithfully. All cues were presented for 1,000 ms. Trials of all blocks had the same sequence and timing, differing only in the shape of the image cue (dot, line, or cross). In precued blocks, participants first saw a cue indicating which image(s) to rate, then the images, then a neutral dot cue. In postcued blocks, participants first saw a neutral dot cue, then the images, and then the cue indicating which image(s) to rate.

Images were presented for 200 ms in their original resolution (500 px × 400 px, about 10.4 deg × 9.3 deg). The short 200-ms presentation duration, well below the minimum reaction time for an eye movement (~250 ms), was chosen to avoid eye and head movements during the presentation. Thus, the four images were at symmetric eccentricities, to avoid driving attentions to whichever image is closest to fixation. Subjectively felt pleasure from an image is reported reliably after presentation durations as short as 50 ms (e.g., Brielmann & Pelli, 2018; Forster, Leder, & Ansorge, 2016; Schwabe, Menzel, Mullin, Wagemans, & Redies, 2018). Participants were instructed to “rate how much pleasure you felt from this image (1–9).” It was emphasized that the content of the pictures was irrelevant, and that there were no right or wrong answers (see Supplementary Material for verbatim instructions). Participants used the keys 1–9 on a standard keyboard to report pleasure from 1 (*no pleasure at all*) to 9 (*very intense pleasure*).

Participants first completed eight blocks of the main experiment (Fig. 1a). A cue pointing to either one or all of the screen quadrants indicates whether to rate the pleasure of one of the presented images, or the combined pleasure of all images. We will refer to ratings of one image among three others as *1-of-4* ratings, and to ratings of the combined pleasure of all four images as *4-combined* ratings.

In the final baseline rating block (Fig. 1b), only one image appears, at a random location. Each image was presented once in the baseline block. We will refer to the ratings of the final block as *baseline* ratings.

Participants first practiced with six training trials, three precued and three postcued, including cues to each of the four quadrants of the screen in 1-of-4 trials, and one precued and one postcued 4-combined trial. After having the opportunity to ask the experimenter questions, observers completed eight rating blocks. Half of the rating blocks showed precues, and half postcues. Precued and postcued blocks were shown in alternation. Whether a participant started with precues or postcues was chosen randomly. In each block, each image was shown once in a random location as target, and once as part of a 4-combined trial (total *n* = 45 per block). At the beginning of each block, participants were told whether the cue would appear before or after the images. Finally, participants completed the baseline rating block. They were then thanked, debriefed, and reimbursed.

We assessed whether the ratings of a participant or for a particular image changed during the time course of the experiment to rule out sequence effects (see Supplementary Material). This also rules out the possibility that baseline ratings at the end of the experiment were systematically corrupted by such sequence effects.

### Analysis

Raw data and analyses files are available (https://github.com/aenneb/tracking4pleasures). All data processing and analyses were performed with MATLAB Version R2018b.

As a measure of accuracy, we calculated the difference between predicted and observed rating per trial. The prediction is produced by the appropriate “faithful” model for the trial type. The faithful predicted rating for a 1-of-4 trial is the baseline pleasure of the target image. Similarly, the faithful prediction for a 4-combined trial is the arithmetic mean of the baseline pleasures of all images displayed in that trial. To assess accuracy, we averaged the prediction error per target baseline pleasure (1, 2, … 9) for 1-of-4 trials. For 4-combined trials, there are many more faithful predictions (from 1 to 9 in 0.25-point increments). To obtain a comparable prediction error statistic for 1-of-4 and 4-combined trials, we created bins of one-point width for 4-combined trials. That is, errors for 4-combined ratings of trials with faithful predictions of, for instance, 1.0, 1.25, 1.5, and 1.75, were averaged into one single bin. We calculated the average error per predicted rating (1-of-4 trials) and per predicted rating bin (4-combined trials) for each participant separately.

We here compare three alternative models to explain the 1-of-4 ratings. Each is listed below along its model equation. We provide equations and model fits for an additional five models in the Supplementary Material.

1. *Faithful model*: The observer reports the baseline pleasure of the target image

1 $$ \hat{P}={P}_t, $$

where $\hat{P}$ is reported pleasure, *t* is the target location (1 to 4), and *P*_*t*_ is the target’s baseline pleasure. *P*_1_ is the upper left image’s baseline pleasure, *P*_2_ the upper right’s, *P*_3_ the lower left’s, and *P*_4_ the lower right’s. Image *t* is the target, and the rest are distractors.

2) *Average-biased model*: The observer’s report of the target pleasure is a weighted average of the target baseline pleasure and the mean baseline pleasure of the displayed images:

2 $$ \hat{P}=\left(1-w\right){P}_t+w\overline{P}, $$

where 0 ≤ *w* ≤ 1 and $$ \overline{P} $$ = $$ \frac{\sum_1^4{P}_i}{4} $$.

3) *High-pleasure attenuation model*: The observer reports the baseline pleasure of the target image if it is below a threshold *P*_beau_, but if it exceeds the threshold, the excess is attenuated:

3 $$ \hat{P}=\Big\{{\displaystyle \begin{array}{ll}{P}_t& \mathrm{if}\ {P}_t<{P}_{\mathrm{beau}}\\ {}{P}_{\mathrm{beau}}+g\left({P}_t-{P}_{\mathrm{beau}}\right)& \mathrm{otherwise}\end{array}}\operatorname{}, $$

where 0 ≤ *g* ≤ 1. Suggested by Brielmann and Pelli (2017).

For 4-combined trials, we considered the following three models to account for the ratings:

1. *Faithful averaging model:* The observer reports the arithmetic mean of the baseline pleasures of the displayed images:

4 $$ \hat{P}=\overline{P}, $$

where $$ \overline{P} $$ = $$ \frac{\sum_1^4{P}_i}{4} $$.

2) *Linear transform*: The observer reports a linear transform of the arithmetic mean of the baseline pleasures of the four presented images:

5 $$ \hat{P}=a+b\overline{P}. $$

3) *High-pleasure attenuation*: The observer reports the arithmetic mean of the baseline pleasure of all displayed images if that mean is below a threshold *P*_beau_; otherwise, the excess above threshold is attenuated in the report:

6 $$ \hat{P}=\Big\{{\displaystyle \begin{array}{ll}\overline{P}& \mathrm{if}\overline{P}<{P}_{\mathrm{beau}}\\ {}{P}_{\mathrm{beau}}+g\left(\overline{P}-{P}_{\mathrm{beau}}\right)& \mathrm{otherwise}\end{array}}.\operatorname{} $$

All these models aim to explain the relation between mean pleasure ratings on baseline, 1-of-4, and 4-combined pleasure trials. They do not refer to variance. We explore the modeling of rating variances in the Supplementary Material.

We performed leave-one-out cross-validation (LOOCV) for each individual participant separately. We fit the various model equations listed above. We assess the error in predicting each trial by a model fit to the rest of the trials. We used the built-in MATLAB function fmincon() to fit the models. The cost function for the minimization problem was the root mean square error (RMSE) between model predictions and observed values for ratings on individual trials. For each model and each observer, we calculated the average RMSE between model predictions and observed rating in the left-out test trials. Last, we calculate the mean RMSE per model across participants as well as the standard error of this mean. All reported correlations are Pearson’s correlations.

## RESULTS

### Prediction error in various trial types

Here, we focus on prediction *error*—namely, the difference between a participant’s single-image or combined-image rating and that predicted by their baseline ratings for the same images. Average 1-of-4 ratings of the target are slightly lower than the baseline rating. The mean error is −0.26 points when precued and −0.42 (*p* = .043) when postcued. In 4-combined trials, the mean error is −0.38 in precued and −0.43 points in postcued trials. Mean error is no different for 1-of-4 than for 4-combined ratings in either cueing condition, and does not differ between precued and postcued 4-combined trials. On a participant-by-participant basis, errors on precued trials are highly correlated with errors on postcued trials for both 1-of-4 and 4-combined trials, both *r* ≥ .82 and *p* < .001. Errors for 1-of-4 and 4-combined trials are unrelated, both *r* ≤ .12 and *p* ≥ .559.

**Fig. 2:**
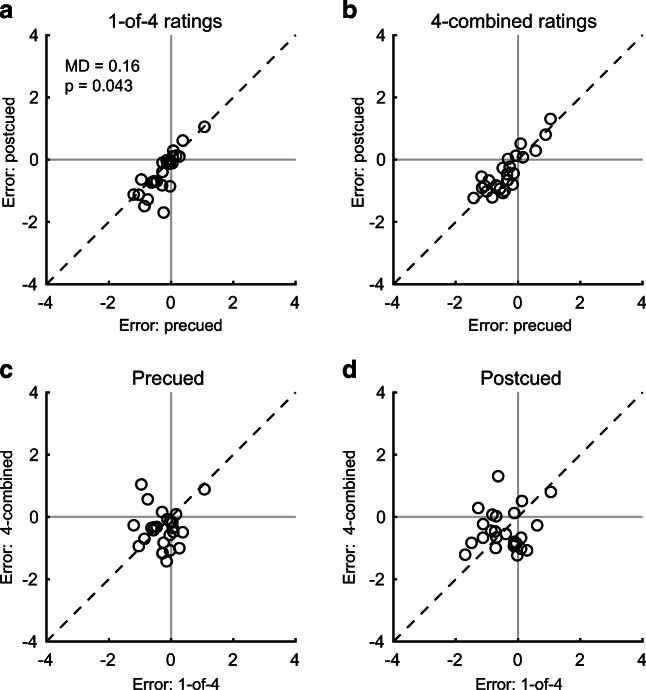
Scatterplots of mean faithful-prediction error per participant (data minus prediction). Light gray lines indicate zero error. The dashed black line is the equality line. Each point indicates the average error across predicted ratings for one participant. **a** Relation between errors in precued and postcued trials for 1-of-4 ratings. **b** Relation between errors in precued and postcued trials for 4-combined ratings. **c** Relation between errors for 1-of-4 and 4-combined ratings in precued trials. **d** Relation between errors for 1-of-4 and 4-combined ratings in postcued trials

Figure 2 illustrates the above findings. Relative to postcuing, allowing participants to preallocate their attention to the relevant image improves faithful-prediction accuracy for their 1-of-4 ratings (see Fig. 2a). Unlike that, faithful-prediction accuracy of 4-combined ratings is independent of cue timing (see Fig. 2b). At the same time, people were just as unbiased in their 1-of-4 ratings as in their 4-combined ratings. In addition, errors in precued trials are strongly positively correlated with errors in postcued trials of the same type (see Fig. 2a–b), but errors in 1-of-4 trials are unrelated to errors in 4-combined trials (see Fig. 2c–d).

We also explored whether the repeated presentation of each image influenced pleasure ratings. Overall, habituation or mere exposure effects were minimal (see Supplementary Material). Therefore, we do not include sequence effects in our models.

### People can tell the pleasure of any of four images if the target location is known in advance

We fit eight models to each participant’s 1-of-4 ratings: faithful, averaging, average-biased, weighted-average indexed by position, weighted-average-biased, linear, weighted-average indexed by pleasure, and high-pleasure attenuation. To avoid overfitting, we perform LOOCV with RMS error as the statistic to assess the goodness of fit of the eight models.

**Fig. 3:**
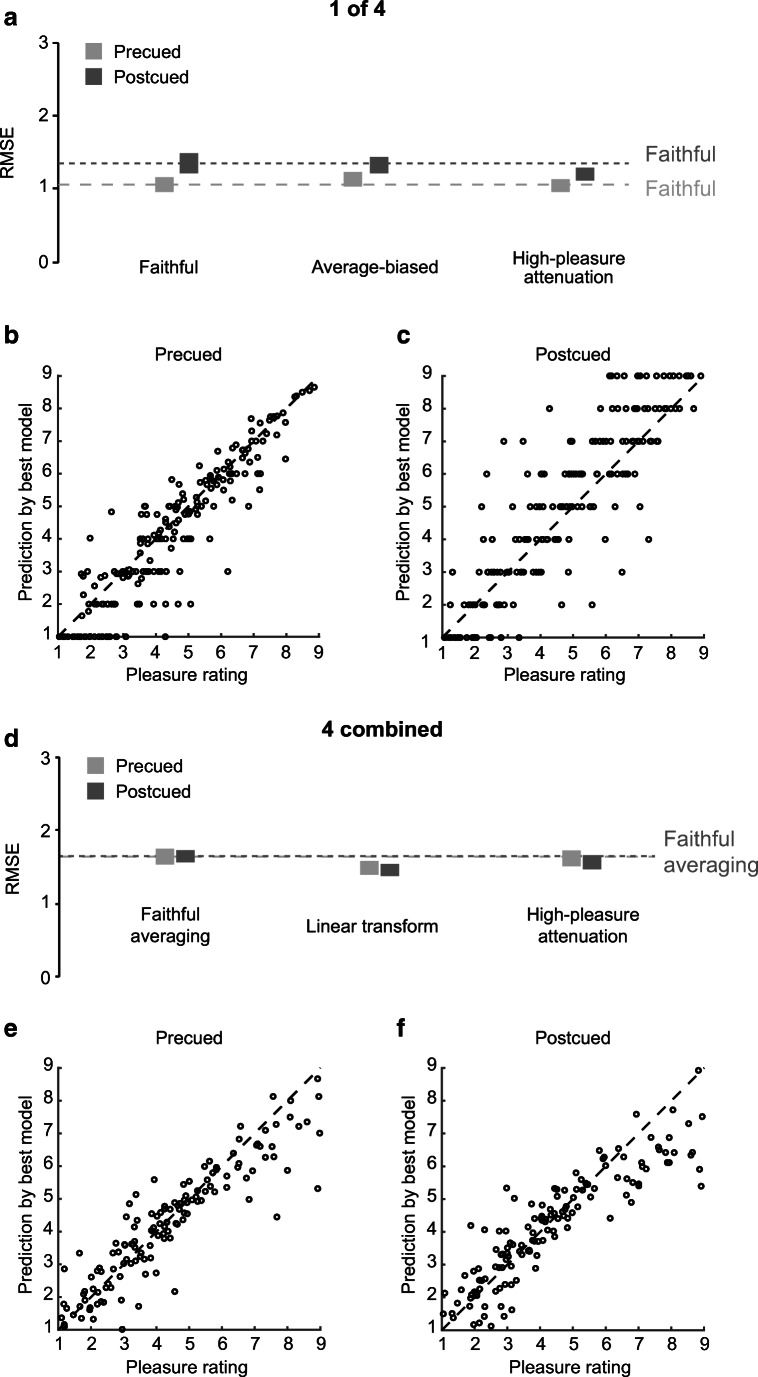
Model fits. **a, d** For each model: average root-mean-square error (RMSE) between ratings and model predictions across LOOCV iterations and participants. The ratings are precued (light gray) and postcued (dark gray) trials for 1-of-4 (**a**) and 4-combined trials (**d**). Bars represent ±*SEM*. The dashed lines indicate the average RMSE for the faithful model for precued (light gray) and postcued trials (dark gray). Models are described in the Methods section. **b, c, e, f** Scatterplots of data (horizontal axes) versus predictions of the best-fitting model (vertical axes) for precued (**b, e**) and postcued (**c, f**) trials for 1-of-4 (**b, c**) and 4-combined ratings (**e, f**). Dashed lines represent equality. **b, c** Each data point represents the average observed and predicted rating for one participant for a 1-point range of average 1-of-4 pleasure across presented images. **e, f** Each data point represents the average observed and predicted rating for one participant for a 1-point range of average 4-combined pleasure across presented images. This means that 4-image trials whose average 1-of-4 pleasures were, for example, 4.25 and 4.75 contributed to the same data point, and trials with averages of 4.25 and 5.25 to separate ones

As illustrated in Fig. 3a, the parameter-free faithful model provides the best fit for the precued results: Its RMSE is no greater than the RMSEs of the alternative models, yet they have additional free parameters. Critically, this finding is robust across participants, with the faithful model having least (or tied) average RMSE in 23 out of 25 participants (see Supplementary Material). In contrast, for postcued trials, the faithful model fits less well than the high-pleasure attenuation model. The high-pleasure attenuation model also outperforms the average-biased model in most participants (18 of 25; see Supplementary Material).

The better fit of the high-pleasure attenuation model in postcued trials is in line with the fact that the errors were, on average, greater and more negative in postcued compared with precued 1-of-4 trials. The average parameter values for the best-fitting model across LOOCV iterations for the high-pleasure attenuation model were a high pleasure threshold of 4.6, *SD* = 1.8, min = 1.0, max = 7.5, and a gain of 0.5, *SD* = 0.3, min = 0.0, max = 0.9. Figures 3b–c compare the best-fitting models’ predictions with the data for precued and postcued trials, respectively.

The fact that the high-pleasure attenuation model fits the postcued data best illustrates that the increasing divergence from single-pleasure ratings is mostly due to lower ratings for otherwise highly pleasurable images in postcued trials. The average observed threshold pleasure above which pleasure ratings are lower than single-pleasure ratings lies around the midpoint of the pleasure scale. This result mirrors the ones of an earlier study, where an added task had an increasingly detrimental effect on the pleasure experienced from images otherwise receiving above-midscale pleasure ratings (Brielmann & Pelli, 2017).

### When combining, people report a linear transform of the 4-image mean pleasure

Analyses of trials in which observers were cued to report the combined pleasure of all images followed the same logic as for 1-of-4 trials. Here, we tested the performance of the following three models: faithful averaging, linear transform, and high-pleasure attenuation.

As illustrated in Fig. 3d, the linear transform model provided the best fit to the data for both precued and postcued trials. Figures 3e–f compare the model predictions to the data. Inspection of the average best-fitting parameters across LOOCV iterations per participants reveals two apparently distinct groups of observers. For both precued and postcued trials, the mean sum squared distance of *k* means cluster analyses confirmed that the parameter distribution is split into two distinct clusters. For both precued and postcued trials, 22 out of 25 observers fall into the first cluster whose centroid parameters are *a* = −1.4 and −1.2, for precued and postcued trials, respectively, and *b* = 1.2 and 1.1. Thus, participants in the first cluster tend to report about 1 point lower combined pleasure than predicted by faithful averaging, but that difference shrinks at higher predicted pleasure. The second, much smaller cluster shows the reverse pattern with *a* = 2.4 and 2.5, and *b* = 0.4 and 0.5; their reports lie above the faithful prediction, and again the difference shrinks at higher predicted pleasure. All but two participants were part of the same cluster (1 or 2) when considering precued versus postcued trials.

### Rating variability is greater in postcued than in precued trials, but unchanged between rating 1-of-4 and 4-combined

**Fig. 4:**
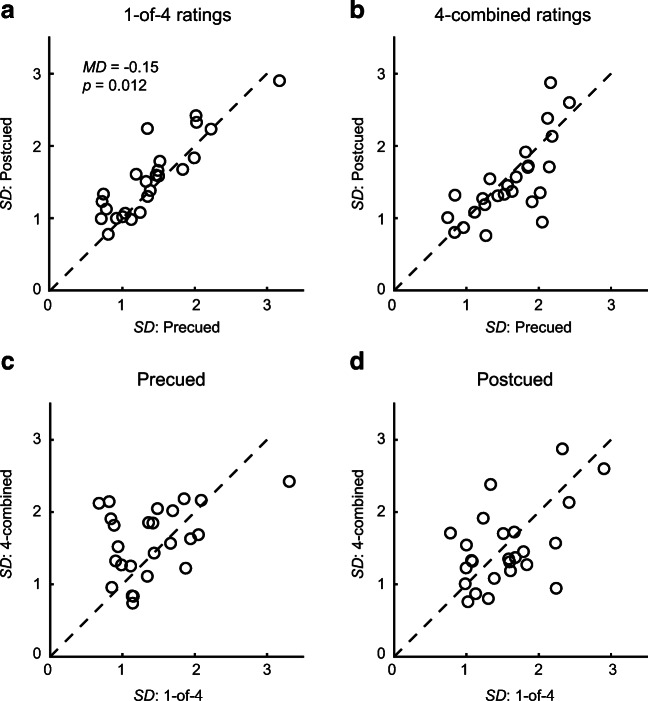
Scatterplots of average pleasure rating standard deviations (*SD*) per participant. Dashed black lines are equality lines. Each point refers to the average standard deviation across expected ratings for one participant. **a** Relation between standard deviations in precued and postcued trials for 1-of-4 ratings. **b** Relation between standard deviations in precued and postcued trials for 4-combined ratings. **c** Relation between standard deviations for 1-of-4 and 4-combined ratings in precued trials. **d** Relation between standard deviations for 1-of-4 and 4-combined ratings in postcued trials

Across participants, the standard deviations for the different trial types ranged from 1.4 to 1.6. Like average errors, standard deviation did not differ between trial types, with the exception that variability was higher in postcued compared to precued trials (*p* = .012, *MD* = 0.15). Figure 4 illustrates these findings. This pattern mirrors our earlier finding that rating variability for 1-of-2 does not differ from variability of 2-combined pleasure ratings (Brielmann & Pelli, 2020). This is still somewhat surprising given the fact that one would expect a reduction of variability due to averaging across not wholly correlated stochastic estimates. The fact that we again found no variance reduction for combined ratings further supports the interpretation that pleasure rating variance in such tasks is dominated by late noise.

Like average errors, standard deviations of pleasure ratings were strongly correlated between precued and postcued trials for both 1-of-4, *r* = .88, and 4-combined ratings, *r* = .73, both *p* < .001. In contrast to errors, however, standard deviations were also correlated between 1-of-4 and 4-combined ratings for both precued, *r* = .40, *p* = .048, and postcued trials, *r* = .52, *p* = .008. Thus, more so than accuracy, rating variability was stable within a given observer across trial types.

## DISCUSSION

Here, we probed how many pleasures people can track, expanding previous findings on tracking the pleasure of two images (Brielmann & Pelli, 2020). By presenting four images at a time, we were able to directly test whether subjective pleasure rating of one item in an array exhibits a mean bias like that of the rating of objective object properties like size. We found that the faithful model best predicted 1-of-4 pleasure ratings when participants were precued which image to rate. When postcued, the high-pleasure attenuation model consistently fit the participants’ ratings best. Combined pleasure ratings were best modelled as a linear transform of the average baseline pleasure across the four displayed images.

### Without precuing, people cannot faithfully report one of four pleasures

People were only able to faithfully report the pleasure of one out of four images when a precue allowed them to preallocate their attention to the target image. In postcued trials that require people to encode and retain the pleasure of all four images, people’s pleasure ratings were not faithful. The pleasure of images that were otherwise highly pleasurable (pleasure >4.6, on average across participants) was diminished. This effect does not represent a bias towards the mean—the effect that has been observed when people report other properties of one target among several distractors (e.g., Alwis & Haberman, 2020; Brady & Alvarez, 2011; Haberman & Whitney, 2009; Maule et al., 2014; Zhang & Luck, 2008). Importantly, we did consider the average-biased model, but found that its performance is consistently worse than the one of either a faithful or a high-pleasure attenuation model (see Fig. 3a).

Our results thus differ from the ones reported by Alwis and Haberman (2020) who found that people are biased toward the mean when reporting the pleasure of one image among four. This difference is most likely due to differences between our and their experimental paradigm. Whereas their participants viewed all four images with the target image clearly marked for 1.5 seconds, our participants only got a 0.2 second glimpse at the images and only knew which image would be the target image in precued, but not in postcued, trials. Thus, it could be that pleasure reports are only biased toward the mean after prolonged stimulus exposure and the associated ability of the participant to shift her attention and gaze across the presented images.

An additional difference between Alwis and Haberman’s (2020) and our study is that we randomly combined images from the entire pleasure range, whereas their displays contained only pleasant *or* only unpleasant images. It might therefore also be that pleasure ratings are only biased toward the mean when the display contains extremely positive and negative images. From an ecological perspective this makes sense, because a summary representation of a homogenous array of items, such as a bunch of mostly ripe bananas, in terms of their mean is useful. In contrast, a heterogenous array of items, such as a few unripe and a few rotten bananas, is not well-summarized as a bunch of on-average ripe bananas.

When presented with a set of objects, people can faithfully report the average size, color, and motion direction (e.g., Alvarez & Oliva, 2008; Burr & Ross, 2008; de Fockert & Marchant, 2008; Neumann et al., 2013; Parkes et al., 2001; Watamaniuk, 1993), as well as facial emotion (Fischer & Whitney, 2011; Haberman & Whitney, 2007) and experienced pleasure (Brielmann & Pelli, 2020). Here, we found that people do not faithfully report the average pleasure across four simultaneously presented images as the combined pleasure. Instead, they report a linear transform of the arithmetic average. Most of our participants report about 1 point lower combined pleasure (on 7-point scale) than predicted by faithful averaging, but that difference shrinks at higher predicted pleasure. A much smaller group of participants shows the reverse pattern: Their reports lie above the faithful prediction, and again the difference shrinks at higher predicted pleasure. Our results do not rule out the possibility that people might be able report the average pleasure if asked to do so (see Myczek & Simons, 2008). Instead, we document what kind of summary representation our participants spontaneously report when summarizing their subjectively perceived pleasure across multiple images.

### 1-of-4 and 4-combined ratings have same variance

As in previous studies (e.g., Allik et al., 2013; Ariely, 2001; Chong & Treisman 2005; Brielmann & Pelli, 2020), we found that reporting the average across items instead of the value of one item failed to decrease the variability of reports. This finding strengthens our previous suggestion that the variance of reporting is limited by a late noise that arises after combining the members of the ensemble.

We only observed one change in rating variability: People’s ratings were more variable for postcued than for precued 1-of-4 trials. This result mimics the reduced variance for valid compared with neutral cue trials reported by Zhang and Luck (2008), albeit their model, which would predict a bias toward the mean, cannot explain our rating results. One potential explanation for the increased variance in postcued trials in our experiment would be that participants need to make more random guesses in postcued than in precued trials. However, a model that assumes a constant rate of such random guesses across all trial types, which we present in the Supplementary Material, fits the pattern of observed rating variance well. Therefore, the current results rather suggest the presence of additional late noise in postcued compared with precued 1-of-4 trials. Given that ratings in postcued trials were best described by a high-pleasure attenuation model that previously accounted for effects of an added cognitive task (Brielmann & Pelli, 2017), it seems plausible that retrieving the pleasure of one out of four images in itself represents a cognitively challenging task and that its execution adds additional late noise to the pleasure reports. Alternatively, additional noise might arise during the encoding of the pleasure from four images simultaneously in postcued trials. Perhaps encoding of the three additional images distracts from the task of encoding the pleasure of the eventual target image.

## CONCLUSION

While people can faithfully track two pleasures, they cannot track four. Instead, the subjectively felt pleasure of otherwise above-medium pleasurable images (pleasure >4.5 on a 1–9 scale) is diminished, mimicking the effect of a distracting secondary task. The rating of the combined pleasure of four images is a linear transform of the arithmetic mean of the four baseline pleasures.

### Acknowledgment

This work was supported by NIH Core Grant P30 EY013079.

### Open Practice Statement

The data and materials for all experiments are available (https://github.com/aenneb/tracking4pleasures).

## Supplementary Information

ESM 1 (DOCX 633 kb)
