# Supplementary material for: The pleasure of multiple images
Source: Atten Percept Psychophys. 2020 Nov 17;83(3):1179–88. doi: 10.3758/s13414-020-02175-z (PMC8049891; doi:10.3758/s13414-020-02175-z)
Supplement: Supplementary file 1 — (DOCX 633 kb) [file 13414_2020_2175_MOESM1_ESM.docx]

The pleasure of multiple images

Aenne A. Brielmann & Denis G. Pelli

**SUPPLEMENTARY MATERIAL**

**Verbatim instructions**

**Introduction (screen 1).** On each trial, you will briefly see 4 images, one in each corner of the screen. You will rate how much pleasure you felt from one or all images (1-9). A box will indicate from which image(s) you should report the pleasure. When making your rating, please ignore whether it''s a picture of something good or bad. Just rate how much pleasure you felt, regardless of the goodness or badness of what''s in the picture.

Hit any key to continue.

**Introduction (screen 2).** You will use the keys 1-9 on the keyboard to rate the pleasure you felt. A rating of 1 means that you felt no pleasure at all. A rating of 9 indicates that you felt very intense pleasure. There is no right or wrong answer. Just rate how much pleasure you felt.

Hit any key to continue.

**Introduction (screen 3).** Between the images, in the middle of the screen, you will see a black cross. Always keep your eyes on this cross. Do not let your eyes drift away. Keep your eyes on the cross.

Hit any key to continue.

**Before training trials.** You will now do some training trials to get you used to your task.

Hit any key to continue.

**After training trials.** Thank you. You have finsihed your prectice trials. Please call the experimenter.

Hit any key to continue.

**Before start of the experiment.** You will now start the actual experiment. \n \n'...

'Hit any key to continue.'];

**Before precued blocks.** In this block of the experiment, we will tell you which images to report on BEFORE they appear. Keep your eyes on the cross in the middle of the screen. Do not let your eyes drift away. Remember: Report only how much pleasure you felt, not the image''s goodness or badness. Rate by hitting a key from 1 (no pleasure at all) to 9 (very intense pleasure).

Hit any key to continue.

**Before postcued blocks.** In this block of the experiment, we will tell you which images to report on AFTER they appear. Keep your eyes on the cross in the middle of the screen. Do not let your eyes drift away. Remember: Report only how much pleasure you felt, not the image''s goodness or badness. Rate by hitting a key from 1 (no pleasure at all) to 9 (very intense pleasure).

Hit any key to continue.

**Relation between single-image ratings and standardized ratings**

To test whether our stimulus selection was effective for the participants in the current experiment, we correlated pleasure ratings in the final single-image block with the standardized valence (Kurdi, Lozano, & Banaji, 2016) and beauty (Brielmann & Pelli, 2019) ratings. We found very high positive correlations for both measures: mean *r =*0.79 and *r =*0.77 for beauty and valence respectively, with minimum *r* = 0.48 and *r* = 0.39, and maximum *r* = 0.98 and *r* = 0.93.

**Pleasure ratings are not influenced by sequence effects**

We assessed whether the ratings of a participant or for a particular image changed during the time course of the experiment to rule out sequence effects and therewith also the possibility that baseline ratings at the end of the experiment were systematically corrupted by such sequence effects. As illustrated in **Figure S1**, no major sequence effect were visible either on a participant or an image basis.

*Figure S1.* Boxplots of correlation coefficients for the relationship between pleasure and image repetition across images per participant (top) and across participants per image (remaining rows). Sequence effects were only evident for participant #8 and #14 and the image “Feces 1”; pleasure ratings decreased over the time course of the experiment. Note that the image exhibiting a slight sequence effect here is different from the single image that we found a sequence effect for in our previous study (Brielmann & Pelli, 2020).

**Additional models considered for 1-of-4 ratings**

In addition to the three models presented in the main paper, we also considered the following five alternative models to fit 1-of-4 ratings. For all equations below $\hat{P}$ is reported pleasure, *t* is the target location (1 to 4), and *P_t_* is the target’s baseline pleasure. *P*_1_ is the upper left image’s baseline pleasure, *P*_2_ the upper right’s, *P*_3_ the lower left’s, and *P*_4_ the lower right’s. Image *t* is the target, and the rest are distractors.

1) *Averaging model*: The observer reports the arithmetic mean of the baseline pleasures of the displayed images.

$\hat{P}=P$ (S1)

where $P$ = $\frac{\sum_{1}^{4} P_{i}}{4}$.

Note that the averaging model (Eq. S1) is the special case of Eq. 2 in the main article with *w*= 1.

2) *Weighted-average model indexed by position*. The observer reports a weighted average of the baseline pleasures of the displayed images.

$\hat{P}=\sum_{1}^{4} {w_{i}P}_{i}$ (S2)

where $w_{i}$ is the weight for the image in position *i* (i.e. which quadrant of the display), the weights sum to 1, $\sum_{1}^{4} w_{i}=1$, and are all positive and no greater than 1, $0\leq w_{i}\leq1$ for all *i*. Eq. S1 is the special case of Eq. S2 with *w*_i_ = *w*/4 for all *i*.

3) *Weighted-average-biased model*: The observer’s report of the target pleasure is a weighted sum of the target baseline pleasure and the weighted average baseline pleasure of the displayed images.

$\hat{P}=w_{0}P_{t}+\sum_{1}^{4} {w_{i}P}_{i}$ (S3)

where $\sum_{0}^{4} w_{i}=1$ and $0\leq w_{i}\leq1$ for all *i*.

4) *Linear model*: The observer reports a linear transform of a weighted average of the baseline pleasures of the displayed images.

$\hat{P}=a+b\sum_{1}^{4} {w_{i}P}_{i}$ (S4)

where $w_{i}$ is the weight for image *i*, the weights sum to 1, $\sum_{1}^{4} w_{i}=1$, and are all positive and no greater than 1, $0\leq w_{i}\leq1$ for all *i*.

5) *Weighted-average model indexed by pleasure*: The observer reports a weighted sum of the baseline pleasures of all four images, where the weight of each image depends only on the rank *r* of its baseline pleasure among the images displayed in that trial.

$\hat{P}=\sum_{1}^{4} {w_{r_{i}}P}_{i}$ (S5)

where *r_i_* is the rank (1 to 4) of the image in position *i*, $w_{r}$ is the weight for rank *r*, the weights sum to 1, $\sum_{1}^{4} w_{r}=1$, and are all positive and no greater than 1, $0\leq w_{r}\leq1$ for all *r*.

**Figure S2A** shows the RMSEs of all additional considered models along with the RMSEs for the models considered in the main article.

**Additional models considered for 4-combined ratings**


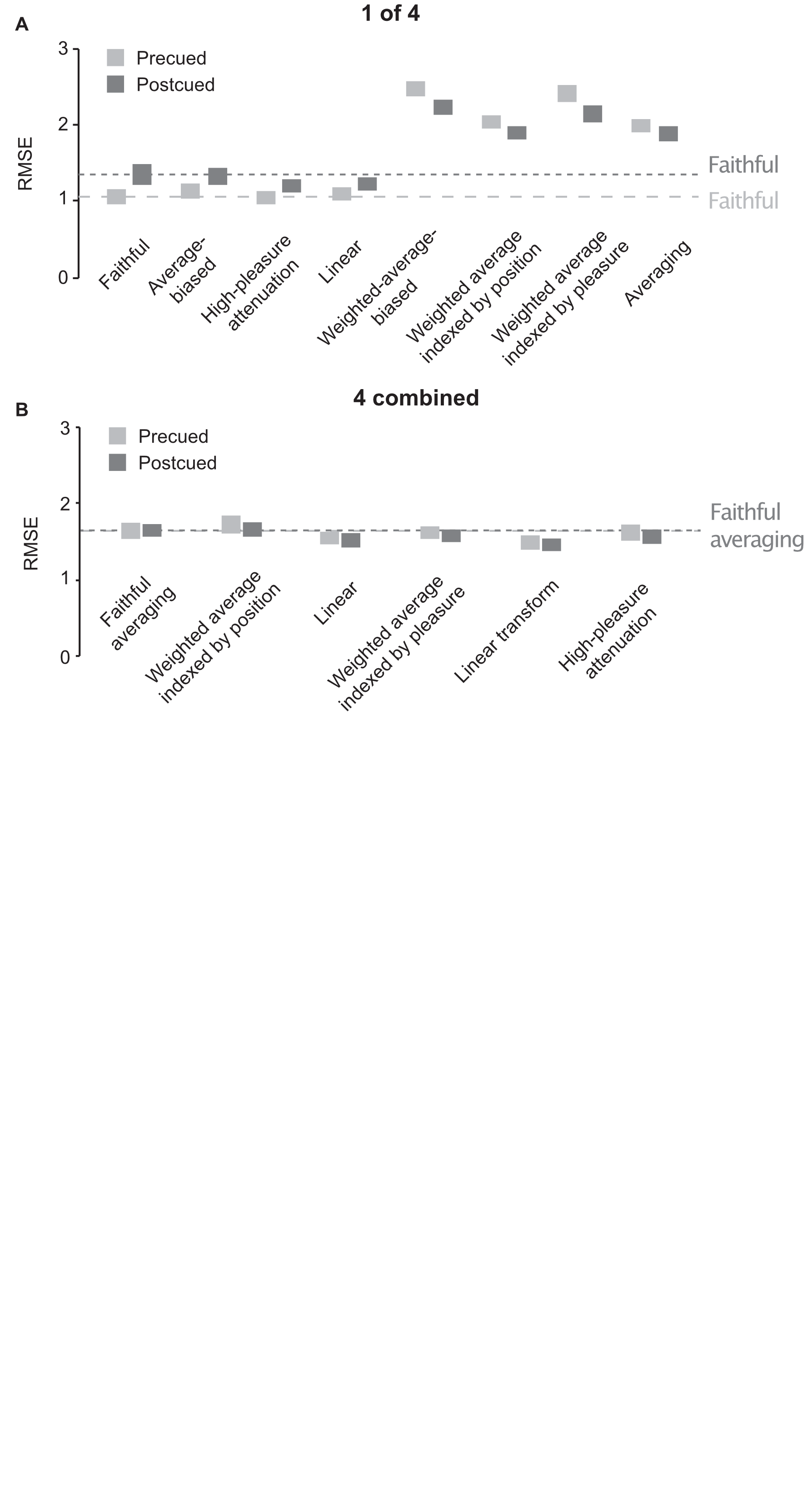


*Figure S2.* **Model fits.** For each model: average root-mean-square error (RMSE) between ratings and model predictions across LOOCV iterations and participants. The ratings are pre- (light gray) and postcued (dark gray) trials for 1-of-4 (A) and 4-combined trials (B). Bars represent ±SEM. The dashed lines indicate the average RMSE for the faithful model for pre- (light gray) and postcued trials (dark gray).

**Consistency of results across participants**

**Figure S3** shows the average RMSE for each of the four best performing models accounting for one-image ratings, **Figure S4** the ones for combined pleasure ratings. Both show that the pattern of results reported in the main manuscript holds true across participants.


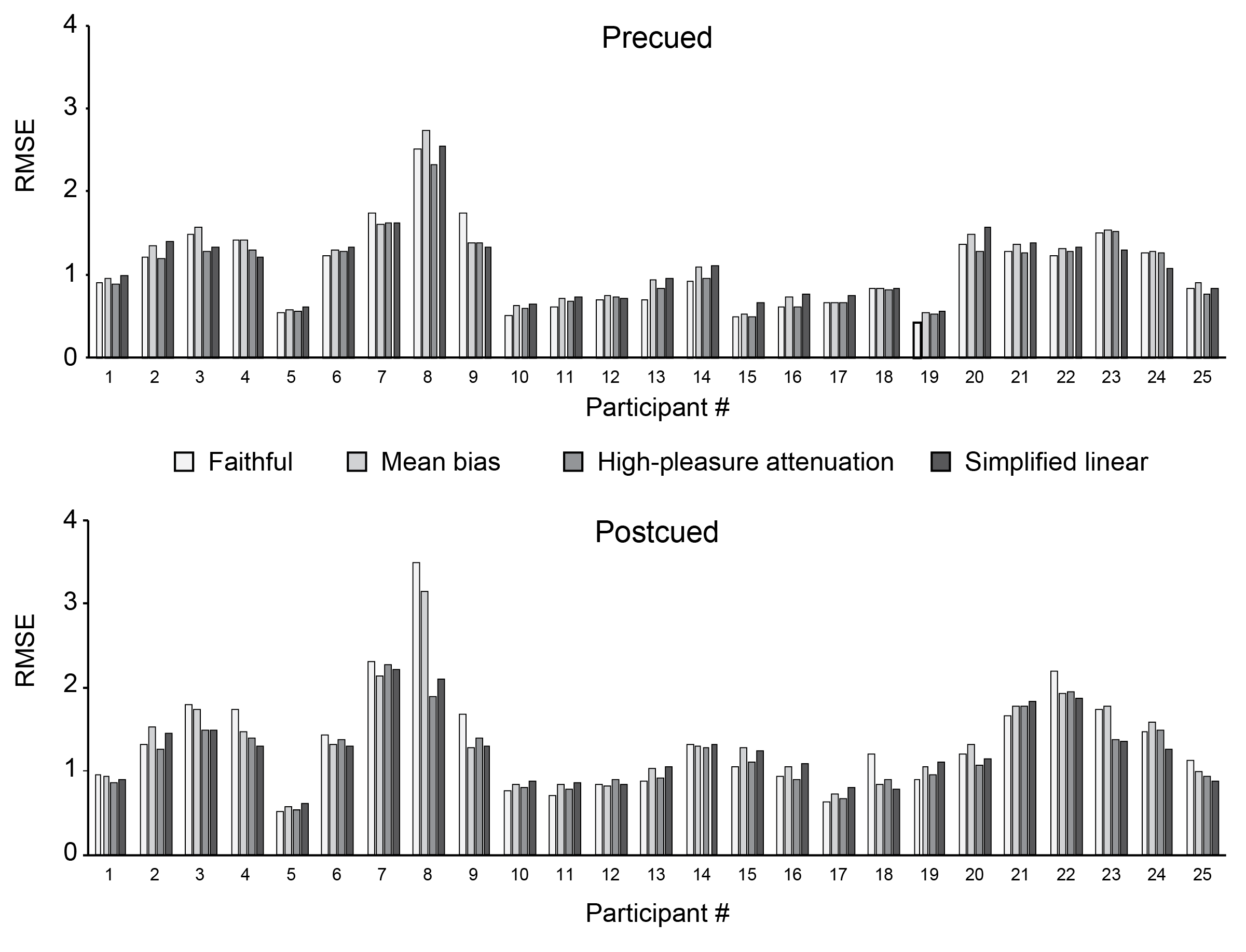


*Figure S3.* Average root mean square error (RMSE) per participant and candidate model based on leave one out cross validation for one-image ratings.


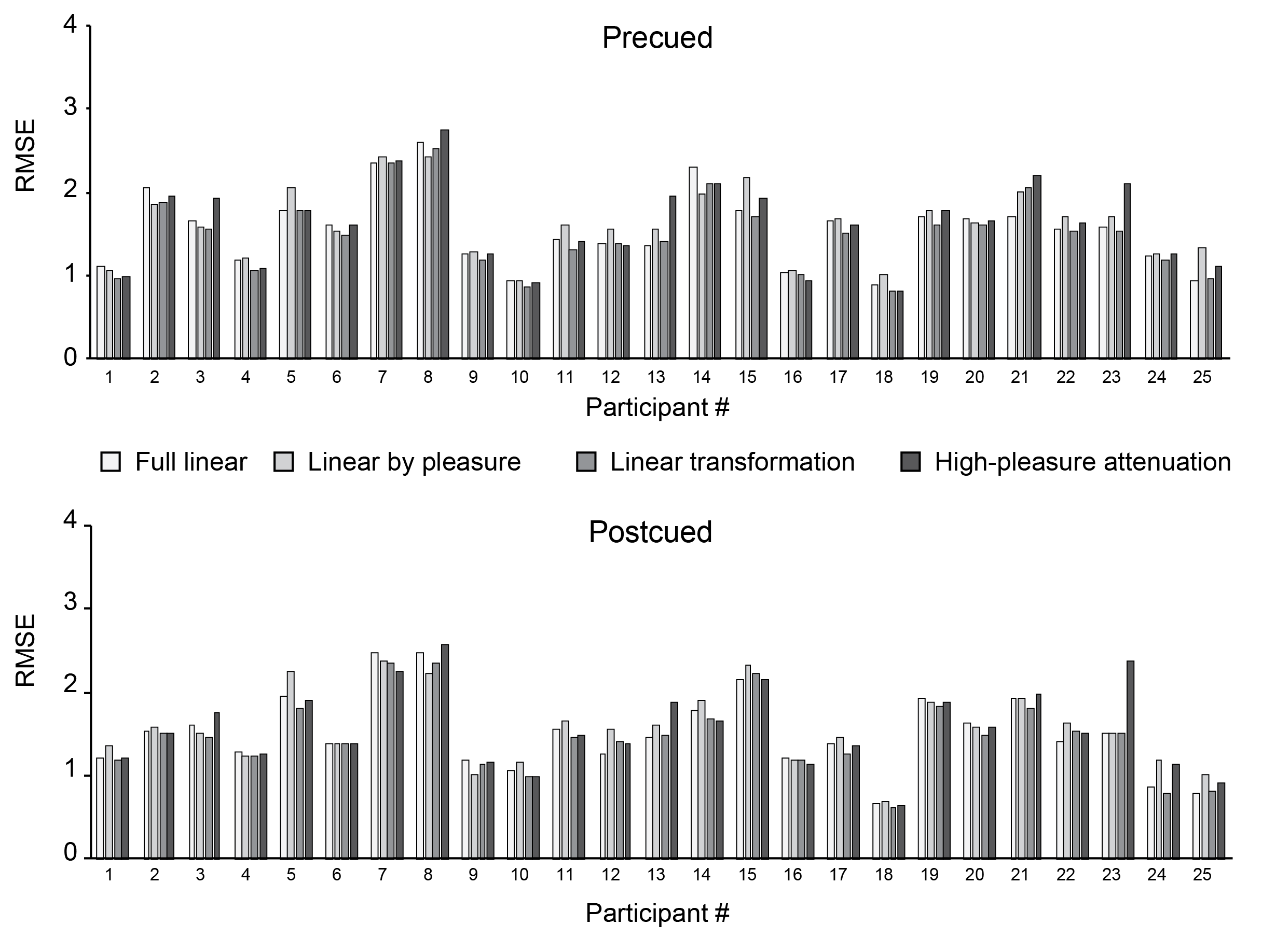


*Figure S4.* Average root mean square error (RMSE) per participant and candidate model based on leave one out cross validation for combined pleasure ratings.

**Accounting for rating variances**

In contrast to the predictions of our models, *SD*s for one- and combined-pleasure ratings were nearly identical. Furthermore, when we looked at the variance of ratings depending on the expected rating, we found that combined-pleasure rating variances were not constant across ratings but followed an inverted u-shaped pattern (see black lines in **Figure S5**). This pattern is identical to the one observed in our previous study where we presented 2 instead of 4 images (Brielmann & Pelli, 2020).

We therefore applied the same *lapse rate model* to the current data that had proven to account for the pattern of *SD*s in our previous article. The lapse rate model builds on the best-fitting models from the main article. In addition, the lapse rate model also contains a lapse-rate component, i.e., it assumes that on a certain proportion of trials, participants experience a lapse of attention and therefore respond with a random number (on the 1-9 scale). Pleasure responses for such lapse trials are sampled randomly from a uniform distribution.

We here assumed the same baseline lapse rate of 4% that we found fit the data for the experiment with 2 images. The effective lapse rate for each participant is the product of 4% and the participant’s average pleasure rating *SD* across target ratings. Here, lapse rates for participants ranged from 2.37% to 13.23% across the different trial types. **Figure S5** shows a good fit between lapse rate model predictions and observed SDs, especially for the inverted-u shaped pattern in combined-pleasure trials.


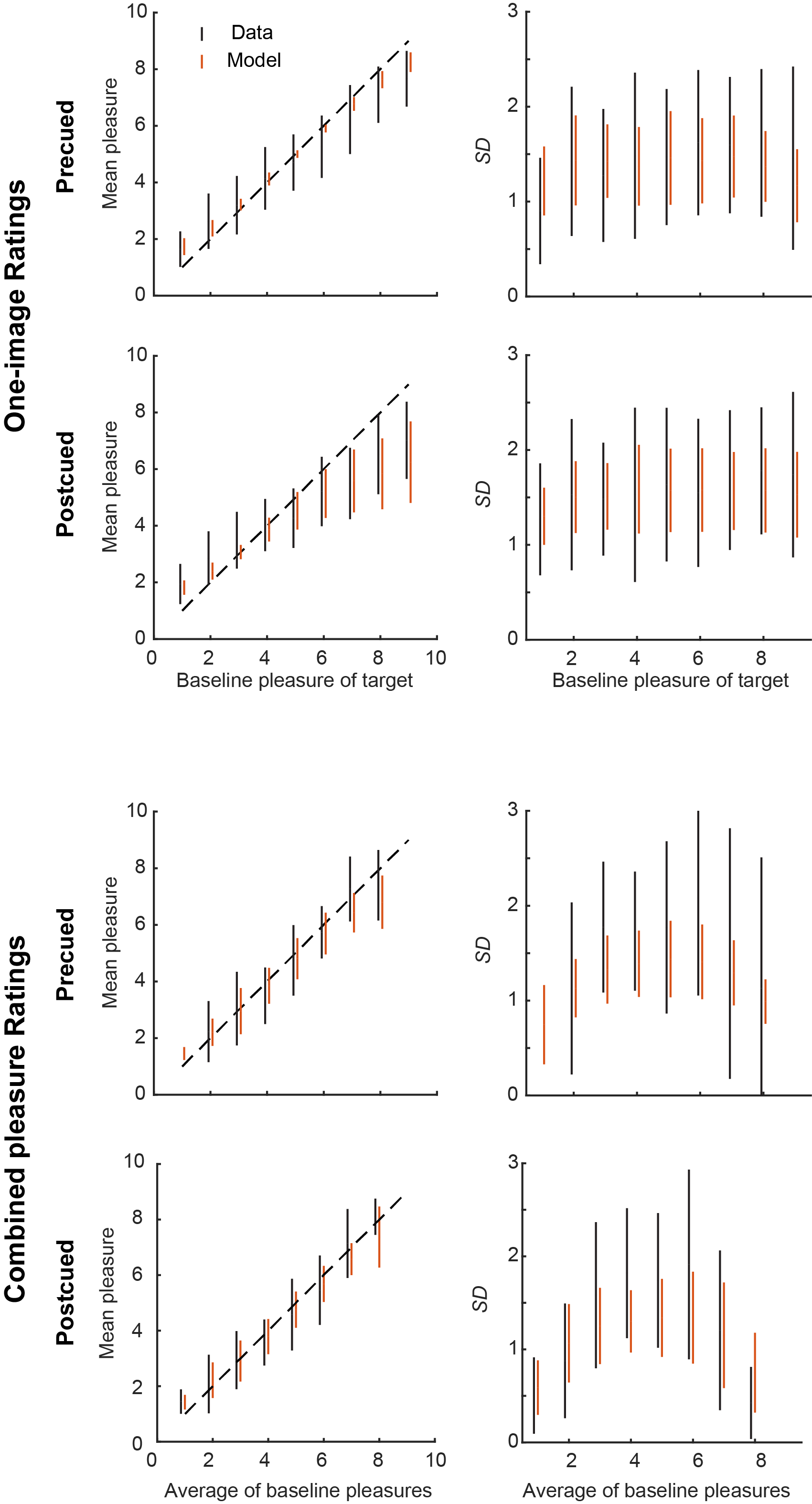


*Figure S5.* Average means (left) and *SD*s (right) per predicted rating for one- (top) and combined-pleasure trials (bottom). Predicted ratings refer to the single-image target pleasure for one-pleasure trials and to the average of all images’ single-pleasures for combined-pleasure trials binned. Data is shown in black, predictions of the lapse rate model in orange. Predictions of the lapse rate model were based on the average of 10 iterations of simulations run separately for each participant using the trial sequence each participant encountered. Error bars represent ±1*SD*.
